# Gastropericardial Fistula Presenting 27 Years after Bariatric Surgery

**DOI:** 10.5811/cpcem.2017.6.34372

**Published:** 2017-10-06

**Authors:** Rebecca A. Martin, Brian Reuhland, Lynn S. Carlson, Michael Love, Robert A. Maxwell, Jessica S. Whittle

**Affiliations:** * The University of Tennessee Health Science Center College of Medicine at Chattanooga, Erlanger Medical Center, Department of Emergency Medicine, Chattanooga, Tennessee; † Erlanger Medical Center, Tennessee Interventional and Imaging Associates, Chattanooga, Tennessee; ‡ The University of Tennessee Health Science Center College of Medicine at Chattanooga, Erlanger Medical Center, UT Erlanger Cardiology, Chattanooga, Tennessee; § The University of Tennessee Health Science Center College of Medicine at Chattanooga, Erlanger Medical Center, Department of Surgery, Chattanooga, Tennessee

## CASE PRESENTATION

**Image 1:**
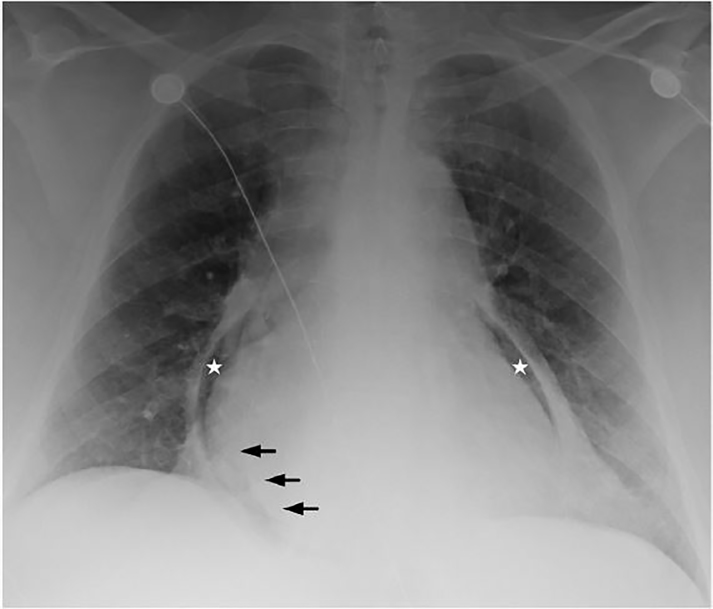
Portable anterior-posterior chest radiograph reveals pneumopericadium (stars) and dependent fluid (arrows) between myocardial and pericardial fat.

**Image 2:**
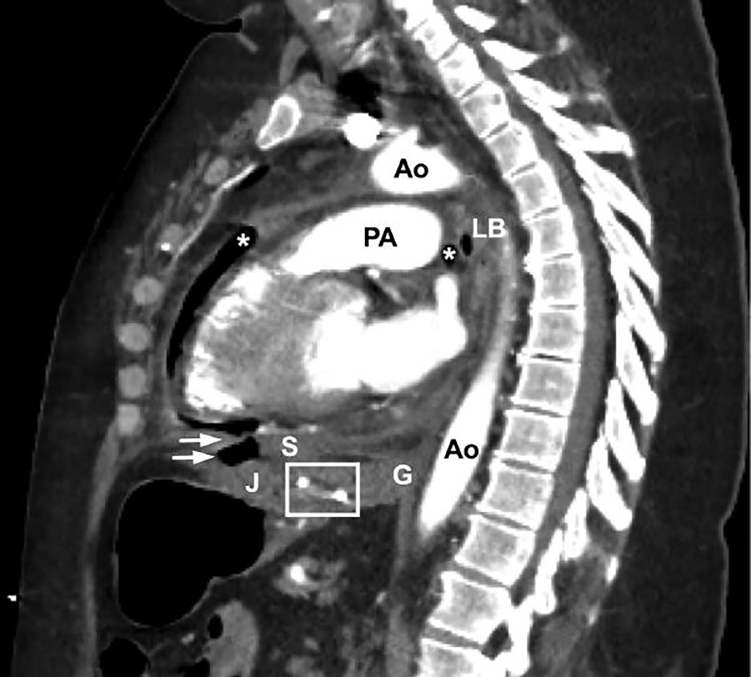
Computed tomography arteriography demonstrates pneumopericardium (*), the gastrojejunal anastomotic ring (box), and air adjacent to the inferior portion of the diaphragm, with notable disruption and thinning of the diaphragmatic contour (arrows). *Ao*, aorta; *G*, gastroesophageal junction; *J*, jejunum; *LB*, left mainstem bronchus; *PA*, pulmonary artery; *S*, stomach.

A 52-year-old female without cardiac disease who had undergone bariatric surgery 27 years prior, presented with three days of worsening chest and epigastric pain. A prehospital electrocardiogram (ECG) was concerning for an ST elevation myocardial infarction (STEMI). Vital signs included a blood pressure of 73/20 mmHg, a heart rate of 113 beats per minute, and tachypnea. On physical exam, the patient was an afebrile, diaphoretic, obese female with epigastric tenderness. Laboratory analysis revealed a troponin of 1.01 ng/mL, a bicarbonate of 12 mmol/L, and a white blood cell count of 39 k/mm^3^. The chest radiograph (Image 1) revealed pneumopericardium and a pericardial effusion. Computed tomography angiography (Image 2) demonstrated extensive inflammation at the cardiac base with thinning of the underlying tissue in proximity to the gastrojejunal anastomosis. Emergent surgery confirmed a gastropericardial fistula, attributed to the failure of a synthetic anastomotic ring. A rising troponin and persistent inferolateral ST elevation on ECG prompted percutaneous coronary intervention, which revealed no stenosis. Despite initial clinical improvement and discharge from the hospital to a rehabilitation facility, the patient’s course was complicated within a few days by recurrent septic shock, pericardial tamponade, and multi-organ failure. Ultimately, her family elected to withdraw care.

## DISCUSSION

Gastropericardial fistula is an uncommon, late complication of gastric or esophageal surgery, including bariatric procedures, with an average of seven years separating surgery and presentation.^1^–^3^ Less common causes include peptic ulcer disease and malignancy.^4^ To our knowledge, this case represents the longest documented time period between surgery and diagnosis.^1^ While gastropericardial fistulae are rare, many of the associated surgical procedures are considered routine.^1^–^3^ Patient presentation is variable, often resembling common pathology: chest pain, septic shock, cardiac tamponade, and STEMI.^1^–^6^ Pneumopericardium on imaging studies is a key finding.^2^,^5^ Successful treatment requires rapid diagnosis, hemodynamic resuscitation, antibiotics, and immediate surgical intervention.^1^ Nonetheless, mortality estimates are high, ranging between 12% and 85%.^1^,^6^

**CPC-EM Capsule**

What do we already know about this clinical entity?

As bariatric surgery has become more routine, rare complications such as enteropericardial fistula may also become more common and mimic other chief complaints.

What is the major impact of the image(s)?

Imaging is the gold standard diagnostic tool for enteropericardial fistula guiding both emergency department and surgical management.

How might this improve emergency medicine practice?

When the presentation is inconsistent with the working diagnosis we must broaden our differential, be aware of anchoring bias, and obtain ancillary information.
